# A narrative review on greater trochanteric pain syndrome: diagnostic imaging and non-surgical treatments

**DOI:** 10.1007/s12306-025-00924-7

**Published:** 2025-09-19

**Authors:** D. Donati, R. Tedeschi, P. E. Garnum, F. Vita, L. Tarallo, C. Faldini, F. Catani

**Affiliations:** 1 University of Modena and Reggio Emilia, Modena, Italy; 2 Policlinico di Modena, Modena, Italy; 3 Independent researcher, Bologna, Italy; 4 Istituto Ortopedico Rizzoli, Bologna, Italy

**Keywords:** Greater trochanteric pain syndrome, Gluteal tendinopathy, Ultrasound imaging, Non-surgical therapy, Extracorporeal shock wave therapy (ESWT)

## Abstract

**Background:**

Greater trochanteric pain syndrome (GTPS) is a common cause of lateral hip pain, primarily affecting middle-aged women. It involves tendinopathy or tears of the gluteus medius and minimus tendons, often misdiagnosed as trochanteric bursitis. Accurate diagnosis and management require thorough clinical assessment and diagnostic imaging.

**Methods:**

This review examines the pathogenesis, clinical examination, and diagnostic tools like ultrasound (US) and magnetic resonance imaging (MRI) for GTPS. A narrative literature was conducted from May 2002 to February 2024 using PubMed. A total of 85 articles were reviewed, with 56 included, focusing on conservative and interventional treatments such as physical therapy, extracorporeal shock wave therapy (ESWT), corticosteroid injections, and platelet-rich plasma (PRP).

**Results:**

Non-surgical interventions showed variable efficacy. ESWT provided significant long-term pain relief, while corticosteroid injections offered short-term benefits that diminished over time. PRP injections demonstrated sustained improvement. US-guided procedures were found superior in precisely targeting anatomical structures.

**Conclusions:**

GTPS remains a challenging, often chronic condition. Non-surgical approaches can effectively manage early stages, but persistent cases may require advanced interventional strategies. Further research is needed to standardize treatment protocols, particularly for severe tendinopathy cases.

## Introduction

GTPS represents a complex and often challenging musculoskeletal condition characterized by lateral hip pain and tenderness around the greater trochanteric area. Although trochanteric bursitis was historically considered the primary cause of GTPS, current evidence highlights gluteal tendinopathy—particularly involving the gluteus medius and minimus—as the main driver of symptoms. Tendinopathy/tears of the gluteus medius and minimus tendons as well as abnormalities of the iliotibial band are now recognized as leading causes of symptoms [1–7]. This condition interferes with sleep (side lying), work, physical activity and other common activities of daily living, with a significant impact to the overall quality of life as much as hip joint osteoarthritis (OA) [8]. It is estimated to affect between 10 and 25% of the population in industrialized societies[9], with a predominance on women over men of up to 3–4 times [10–12] The incidence rate in general population ranges from 1.8 to 3.29 per 1000 person-years [13, 14], with the incidence peak occurring between the fourth and sixth decades of life [15, 16]. A higher prevalence can be found in patients with co-existing conditions such as iliotibial band (ITB) tenderness, ipsilateral and contralateral knee osteoarthritis (OA) and low back pain (LBP) [11, 17]. Several studies [10, 12] report that a relevant percentage of patients referred to spine specialists for LBP and degenerative lumbar pathologies are affected by GTPS, meaning that GTPS may be undiagnosed or even misdiagnosed. It is proven to represent a chronic condition in a large number of patients, with symptoms persisting at least in 36% and 29% of the patients, respectively, after 1 and 5 years of follow-up [14]. Many patients with GTPS still suffer from hip pain after 11 years from diagnosis and appear to have a higher risk of developing hip OA than an asymptomatic control group [18] [14]. The greater trochanter consists of four facets: anterior, lateral, posterosuperior, and posterior. The gluteus minimus tendon mainly inserts onto the anterior facet, whereas the anterior and posterior tendons of gluteus medius insert, respectively, onto the lateral and posterosuperior facets. The muscles in the lateral hip region are organized in a superficial layer, consisting of the tensor fascia lata and gluteus maximus muscles, and a deeper layer, the gluteus medius and minimus muscles. The fascia lata overlies the gluteus medius muscle filling up the interval between the tensor fascia lata and the gluteus maximus muscles. It thickens at the level of the greater trochanter becoming iliotibial tract. The iliotibial band and tensor fascia lata work together as a lateral tension band to resist strains over the greater trochanter [19]. The gluteus medius and minimus muscles can be considered the “rotator cuff” of the hip joint [20] as they participate to the abduction mechanism of the hip joint. Their tendons like the supraspinatus and infraspinatus are prone to trigger points, tendon degeneration or tendon failure. Likewise, the subacromial bursa of the shoulder have potential for inflammation as the deep trochanteric bursae in the hip. It has even been postulated that overlying rigid and unyielding structures such as the iliotibial band (ITB) at the hip may cause external impingement or compressive irritation as the acromion and coracoacromial ligament in the subacromial space [21]. Approximately 20 bursae ensure protection from frictional forces between the tendons located in the greater trochanteric area. Some bursae may be acquired from excessive friction or increased hip offset, but the subgluteus minimus, the subgluteus medius and the subgluteus maximus bursae are consistently present in most individuals. The subgluteus minimus bursa is located anterosuperiorly to the greater trochanter, deep to the anterior border of the gluteus minimus tendon as it passes around the anterior aspect of the greater trochanter to its insertion [22]. The most superior bursa, the subgluteus medius bursa, lies deep to the gluteus medius tendon sitting superiorly to the greater trochanter. The deep subgluteus maximus bursa is the largest and often described as the “trochanteric bursa”. This lies lateral to the greater trochanter between the gluteus medius and gluteus maximus tendons.

## Materials and methods

In order to develop a complete and comprehensive review about GTPS, the authors conducted an extensive review of the recent (from May 2002 to February 2024) scientific literature about greater trochanteric pain syndrome, mainly focusing on pathogenesis, clinical examination, sonographic evaluation, and treatment based on conservative and interventional procedures. Research was conducted on PubMed using the following keywords: “Greater trochanteric pain syndrome”, “Trochanteric Bursitis”, “Gluteal tendon tear”, “Gluteal Tendinopathy”, “Hip anatomy”, “GTPS epidemiology”, “hip abductor tendon tears”, “GTPS conservative treatment”, “GTPS surgical treatment”, “GTPS ultrasound”, “GTPS US-guided procedures”, “GTPS injections”.

ll information was collected, screened based on clinical relevance, and synthesized narratively, with a particular focus on ultrasound imaging and non-operative treatment strategies. Given the narrative nature of this review, no formal criteria for study inclusion, exclusion, or statistical comparison were applied, and no flowchart was constructed.

## Presentation

GTPS typically presents as chronic, persistent pain in the lateral hip and/or buttock that is exacerbated by lying on the affected side, prolonged standing or transitioning to a standing position, sitting with the affected leg crossed, climbing stairs, running, or other high impact activities[9] In the acute phase, pain worsen when applying pressure on the affected side, i.e. while lying down, and symptoms may also extend laterally down the thigh or posteriorly into the gluteal region in a non-dermatomal pattern [21]. Associated lateral thigh pain radiation is possible, but rarely below the knee [19, 23].

## Clinical examination

In a recent systematic review [24], the authors assessed 23 full texts, including six studies for review involving 15 tests and 272 participants (314 hips). The most common tests and signs utilized for assessing GTPS are resisted hip abduction test (pain), Trendelenburg sign, GT palpation test (pain), single-leg 30-s resisted stance test (pain), resisted hip external derotation test (pain), resisted hip internal rotation test (pain/weakness) and the hip flexion/abduction/external rotation (FABER) test (pain). Others like hip lag sign (strength), passive hip adduction (pain), hip flexion/adduction/external rotation (FADER) (pain), passive hip internal rotation (pain), passive hip abduction (pain), active hip internal rotation (pain), hip abduction (strength), and passive hip internal rotation (range) are also useful. The authors produced a clinically useful sequenced 2-test cluster to help clinicians diagnose GTPS more easily in patients with lateral hip pain (LHP), without the necessity to perform an extended battery of pain provocation tests. The 2-test cluster consists in GT palpation test and resisted hip abduction test to determine the post-test probability of GTPS diagnosis: a negative GT palpation test followed by a negative resisted hip abduction test significantly reduces the post-test probability of GTPS, and a positive GT palpation test followed by a positive resisted hip abduction test significantly increases the post-test probability of GTPS. Their meta-analysis also convincingly identified that none of the other tests evaluated (Trendelenburg sign, single-leg 30­second stance, resisted external derotation, resisted, FABER), except the resisted hip abduction test, could significantly shift the post-test probability both towards and away from a GTPS diagnosis. The role of resisted external rotation test in shifting post-test probability of a GPTS diagnosis highlighted in a previous meta-analysis [25] is also being redimensioned because of the high risk of bias with potential test-accuracy overestimation, as the advocation of the single-leg 30-s stance test for diagnosing GTPS. Nonetheless, the single-leg 30-s stance test performed after a positive GT palpation test could confirm a GTPS diagnosis if positive, but would not be able to rule out a diagnosis of GTPS when negative.

Fearon et al. [26] selected the FABER test as a key criterion for differentiating lateral hip pathology from hip joint pathology, assessing that soft tissue pathology of the greater trochanter is likely to be present when the FABER test reproduces the patient’s lateral hip pain but the hip is not limited in terms or range of motion (absence of difficulty manipulating shoes and socks). If motion is limited, then hip joint-based pathology cannot be excluded.

A summary of the most relevant clinical tests used in the diagnosis of GTPS is provided in Table 1. This includes palpation, resisted abduction, FABER, and the single-leg stance test, which are the most reliable tools to confirm or exclude the diagnosis in clinical practice. These tests should be interpreted within the context of a comprehensive clinical evaluation.

**Table 1:** Most relevant clinical tests for diagnosing GTPS and their diagnostic features

Test	Description	Diagnostic value	Notes
Greater trochanter palpation [24]	Palpation directly over the greater trochanter	High sensitivity and specificity when combined with resisted tests	First-line test. Positive if localized pain is elicited
Resisted hip abduction [24]	Patient resists abduction from neutral or slight abduction position	Strong predictor for GTPS	Combine with palpation for highest diagnostic yield
FABER test [26]	Flexion, abduction, external rotation of the hip	May indicate soft tissue involvement around greater trochanter	Positive when lateral hip pain is reproduced
Single-leg stance (30 s)[24]	Patient stands on one leg for 30 s without support	Moderate diagnostic value	Useful when combined with palpation and resisted tests
External derotation test [25]	Patient supine, hip flexed at 90°, examiner applies resistance to external rotation	Controversial. Risk of overestimation due to bias	Previously used, now considered less reliable

## Differential diagnosis

Intra-articular sources of hip pain include labral tears, loose bodies, femoroacetabular impingement (FAI), capsular laxity, ligamentum teres rupture, and chondral damage [27]. GTPS and FAI may share a similar clinical presentation, but they do not co-exist frequently, especially in younger patients (under 40 years) with FAI. Conversely, GTPS may represent a hidden cause of hip pain in patients suspected of FAI, particularly in elderly patients (over 40 years) with normal hip anatomy at MRI [15].

Extra-articular sources include stress fractures, piriformis syndrome, and neoplasms. Sources of hip pain that are outside the hip include pathology of the superior gluteal nerve, meralgia paresthetica, lumbar spondylosis, and lumbar radiculopathy. In patients with lumbar spondylosis or lumbar radiculopathy as in patients with GTPS, a limp and hip abductor weakness may be present along with radiating pain [27] that follows a dermatomal distribution. The correct diagnosis can be challenging because these conditions and GTPS may co-exist. A tight ITB can snap over the greater trochanter causing a painful condition called external snapping hip syndrome, especially in younger and active patients [19]. Patients with a history of total hip arthroplasty, especially through an anterolateral approach, may have iatrogenic injury to the abductor mechanism or its innervation [27].

### Imaging

- *Radiography*: radiographs of the pelvis are routinely performed to exclude differential diagnoses such as hip osteoarthrosis, FAI, trochanteric avulsion fracture and periosteal neoplastic lesions or secondary malignancy of the proximal femur [28]. Surface irregularities including calcifications of abductor’s tendons, exostoses or enthesophytes of the greater trochanter can be found; however, these types of findings are not reliable radiological indicators for the diagnosis of GTPS [29].
- *Sonographic Imaging*: US has been reported to have a sensitivity of 79% to 100% and a positive predictive value of 95% to 100% for gluteal tendon tears, but its diagnostic value is highly operator dependent [4]. Bursal effusions can be seen as large anechoic collections. Degenerative changes of the gluteus tendons are represented as tenohypertrophy, heteroechogenicity, neovascularity, and abnormal tendon architecture. Partial-thickness and full-thickness tendon tears are seen directly as hypoechoic or anechoic foci or indirectly as contour defects, through transmission enhancement or edge artefacts [28]. Furthermore, in a cadaveric study [20] US guidance was found to raise the accuracy of GTPS injections at 92% over the 67% accuracy of the landmark-guided approach. Performing interventional procedures with US-guide allows visualization of all the pertinent structures involved in GTPS, the needle tip position, and real-time appreciation of the spread of the injectate, leading to potentially better outcomes. This find confirms in prior studies [30–32], indicating superior accuracy and outcomes improvement for injection procedures when performed with sonographic guidance.
- *MRI*: MRI has a reported sensitivity of 73% and specificity of 95% for diagnosing gluteal tendon tears [4]. Trochanteric bursopathy may be evident as a T2 signal change, but usually it is not an isolate finding. Degenerative changes to the gluteus medius and gluteus minimus tendons can be seen as abnormal T2 signal change with disturbed tendon architecture on T1-weighted imaging. Partial-thickness and full-thickness tears in the hip abductors, as well as muscle atrophy and fatty degeneration, can also be seen on MRI [28]. Grade 3 tendinopathy (partial-thickness tears) is diagnosed via increased signal intensity on T2-weighted MRI scans, and grade 4 tendinopathy (full-thickness tears) is shown as a discontinuity of one or both gluteal tendons [33].

**Fig. 1:**
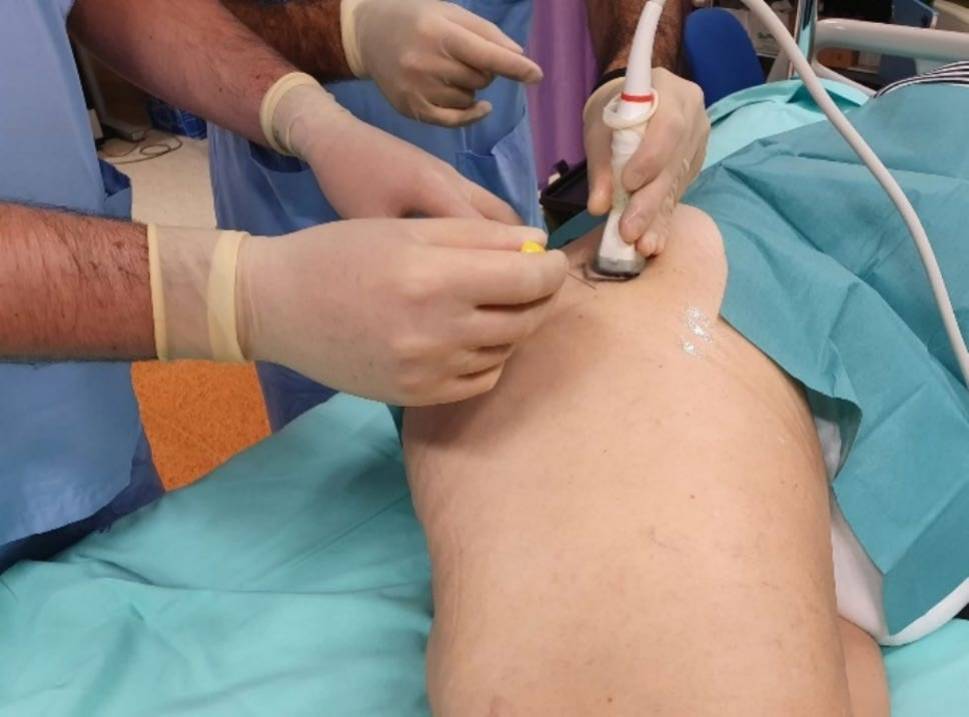
Ultrasound-guided intervention

**Fig. 2:**
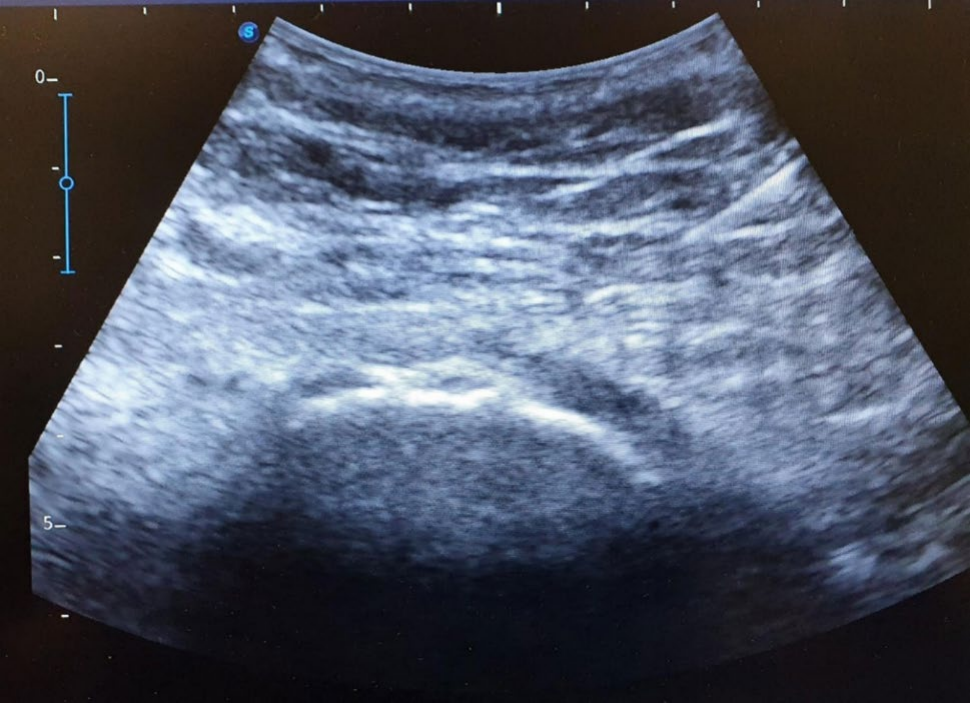
Transverse ultrasound image

Figures 1 and 2 illustrate ultrasound-based evaluation and intervention in GTPS.

Figure 1 shows the clinical setting of an ultrasound-guided injection over the greater trochanteric area, highlighting the operator’s positioning and probe orientation.

Figure 2 shows a transverse ultrasound scan of the greater trochanteric region, where gluteal tendon fibres and adjacent bursal structures can be appreciated. These visual representations aim to enhance understanding of the role of ultrasound in both diagnosis and intervention.

Ultrasound-guided intervention on the greater trochanteric region. The operator is positioning the needle under real-time ultrasound guidance to target pathological tissue with precision.

Transverse ultrasound image of the greater trochanteric area showing the cortical surface of the femur and the hypoechoic changes in the gluteal tendon consistent with trochanteric bursa.

### Conservative treatment

Extracorporeal shock wave therapy; RMS: Roles and Maudsley score; HAM: hip adduction moment; vGRF: vertical ground reaction force; FPI-6: foot posture index; EVA: ethylene–vinyl acetate.

- *Diet and lifestyle:* active or relative rest with reduction in weight bearing load is preferred to complete immobilization to enhance recovery and reduce further complications. Postural education and activity recommendations can assist the patient to control their symptoms. Positions of hip adduction should be avoided to decrease muscular tension and compression across the painful area. When sleeping the patient should seek a softer mattress or use a donut-shaped relief pad when lying on the symptomatic side. The patients should avoid unilateral stance positions where they bear the majority of their weight on the involved extremity and let the hip “hang” or drop to the opposite side. In sitting, crossed-legs position should be avoided particularly when one thigh is flexed and crossing over the uninvolved side. The patients should avoid hills and consciously shorten stride length while walking. Exaggerated frontal or transverse plane pelvic motion inherent to “power or race-walking” should also be minimized in the early phase of treatment. If there is gluteus medius weakness, the patient should use the handrail on the uninvolved side when descending stairs to unload the symptomatic hip’s compressive pain generation [21]. Lastly, weight loss can successfully help in managing the symptoms [34].
- *Foot orthosis:* few articles can be found in the literature about their use for GTPS. The most recent study [35]analysed if medially posted foot orthoses could immediately reduce hip adduction moment and pain in females with GTPS during walking gait. The results show that prefabricated medially posted foot orthosis do not immediately alter gait biomechanics or provide a clinically meaningful reduction in pain.
- *Physical Therapy and home exercise:* in a recent review [36], the authors studied the effects of exercise-based interventions on gluteal tendinopathy. The results indicate that resistance exercise yields superior results in terms of function and severity of symptoms, but not in quality of life in the short and long term when comparing with sham exercises (exercises which do not generate tension in the gluteus medius and minimus muscles). Isometric and kinetic chain exercises, isolated isometric and isotonic exercises, strengthening exercises with progressive load, functional exercises, stretching, and home exercises were compared. All protocols followed a 12-week period based on a daily exercise regimen. All exercise protocols produced positive effects on clinical outcomes such as pain intensity, physical function, perceived change, activity levels, and strength. However, due to heterogeneity among interventions, it remains unclear which specific regimen is superior. Adding education on load management and avoidance of tendon compression to exercise could be an important aspect for the management of gluteal tendinopathy. They also found no difference in reduction of pain intensity between exercise and CSIs, but exercise showed a higher treatment success rate when compared to corticosteroid infiltration in both the short and long term. However, this analysis presented substantial heterogeneity and low certainty of evidence. One high-quality randomized clinical trial [37], however, showed that targeted exercise for gluteus minimus and medius muscles plus education for tendon load management result in a higher treatment success rate when compared to CSI both in the short (8 weeks) and long (52 weeks) term in individuals with gluteal tendinopathy.
- *Extracorporeal Shock Wave Therapy (ESWT):* the use of SWT in gluteal tendinopathy is supported by high level of evidence [4]. In terms of pain relief, one study [38] showed that, compared with CSI, ESWT significantly decreased NRS score at 4 months and 15 months. Good long-term results can be achieved for grade 2 tendinopathy, with improvements maintained over 27 months [39], but there are concerns as to whether this treatment option is suitable for grade 3 tendinopathy. In addition, the lack of a standardized treatment protocol limits the applicability of SWT in the treatment of gluteal tendinopathy.

### Interventional procedures

- *Local anaesthetic (LA) and corticosteroid injection (CSI):* the benefits of CSIs in GTPS are still debated. Different studies found no significant effect on pain reduction and function improvement when comparing CSIs to no treatment both in the short and long term [40], LA + CSI to placebo at 1, 3, and 6 months [41] and no superior clinical benefit of CSI to dry needling at 6 weeks [42]. Conversely, in a systematic review[4]CSI in grades 1–3 tendinopathy was found to have a positive short term (first 4 to 8 weeks) effect allowing remarkable pain improvement, with benefits lasting no longer than 3 to 6 months. It is worth noting that the therapeutic response was less successful after structural abnormalities within the gluteal tendons had developed. Furthermore, the same review included articles that reported significantly improved outcomes up to 104 weeks from baseline. The results aforementioned for short-term effects of CSIs find confirm in recent studies [36, 43]; however, the benefits appear to decrease after 3 to 6 months from initial treatment. Another meta-analysis [43] suggests that CSI is still effective in terms of long-term efficacy at 12 months. The available evidence therefore seems to support the use of CSIs for low- to moderate-grade tendinopathies only for short-term pain relief. It is important to consider risks and local plus systemic side effects linked to local CSI, such as damage to tendinous structures, increased tenocyte senescence with potential long-term degenerative changes in tendon tissue [44], increased collagen disorganization and collagen necrosis leading to an overall negative effect on tendon homeostasis and short-term deterioration in mechanical properties [45]. This taken into account, Jarlborg et al. [46] conducted a secondary analysis of a randomized controlled trial (RCT) to identify clinical predicting factors leading to a positive response to CSI. They found that GTPS patients who present a rapid decrease in pain within 30 min after periarticular injection (both GC + LA and placebo), as well as patients who display a combination of positive resisted external derotation test, FABER test and 30-s single-leg stance test, are more likely to benefit from local GC and anaesthetic injection than the remainder of patients with GTPS. Moreover, the site of injection plays an important role, since evidence shows that corticosteroids injected into the greater trochanteric bursa (aka subgluteus maximus bursa) may be associated with larger and longer-lasting pain reduction over subgluteus medius bursa or non-bursal injections [47, 48].
- *Platelet-rich Plasma (PRP) injection:* a randomized controlled trial [49] showed that a single PRP injection achieved significant better outcomes at 12 weeks when compared to a single CSI, especially at modified Harris Hip Score (mHHS) and Patient Acceptable Symptom State (PASS). In the same study, the benefits in the CSI group started to decrease after 6 weeks to baseline, conversely the PRP injection group consistently improved at 12 weeks. To note that, all the participants in this study had failed previous physical therapy interventions. The same research group then conducted a follow-up study [50] on the same pool of patients. The leucocyte-rich PRP group continued to progress at 2 years of follow-up, with only two patients crossing over to surgery and two patients being lost to follow-up. During the 2 years of follow-up, of the 37 patients in the CSI group, ten remained blinded as treated and 27 crossed to a new formed CSI + LR-PRP group (within this subgroup, three failed the LR-PRP injection and were referred for surgery). The latter improved with the same pattern of progression in outcomes as the LR-PRP group, demonstrating that a prior CSI did not influence the outcome of the LR-PRP injection. The correlation between grade of tendinopathy and outcome was not formally assessed, but interestingly the LR-PRP group had twice the number of grade 3 tendinopathy patients, potentially leading to worse response rate which did not occur.
- Other factors acknowledged to contribute to the variability of the treatment effect are the type of preparation, platelet concentration, activity of PRP (activated vs nonactivated), and frequency of applications. Based on the available literature, a single ultrasound-guided application of nonactivated LR-PRP with a preparation centrifugal force of 1050g to 1250g and a spin time of 14 to 17 minutes should be used [4].
- *Hyaluronic acid injection (HA):* few literature is available on the effect of HA for the treatment of GTPS. A retrospective study [51] confronted the outcomes of three groups: one treated with CSI, one with HA, and one with a combination of both for trochanteric bursitis. The results suggested that HA injection or a combination of HA + CSI might be a valid alternative to LA + CSI alone, showing a larger effect size on pain relief and longer efficacy, and could be used especially in diabetics. Another prospective randomized trial [52] found a non-inferior effect of HA injection to LA + CSI at 1, 3 and 6 months of follow-up, both gaining a significant decrease in VAS pain score, therefore highlighting the possibility of considering HA as an effective alternative therapeutic strategy for patients in whom the treatment with LA + CSI have been unsuccessful or contraindicated.
- *Percutaneous needle tenotomy/Tendon Fenestration/Dry needling:* dry needling is a procedure in which a solid filament needle is used to repeatedly penetrate the skin, subcutaneous area, tendons and muscles to increase blood flow, reduce trigger points, and mechanically disrupt the tissue without using any biological agent [53]. This causes bleeding of the tendon which increases the inflammatory process, induces the release of beneficial growth factor [54], promotes a more ordered collagen formation [55], and more importantly induces change from a chronic non-healing injury into an acute lesion that may have greater healing potential [56]. Needle tenotomy is often performed in conjunction with injections such as PRP, but data on the effect of percutaneous needle tenotomy (tendon fenestration) alone are limited, especially on GTPS. A retrospective cohort analysis [57] concluded that 54% of patients treated using tendon fenestration for grade 2 or 3 tendinopathy of the gluteus medius or minimus tendon reported marked improvement of their symptoms, while another study found that the effect of a single PRP injection plus tendon fenestration was not superior to that of a single PRP injection alone [4].
- One study [42] found no inferiority in pain (numeric pain rating scale 0-10) and function (Patient-Specific Functional Scale) at 6 weeks after treatment between CSI and dry needling, although hips treated in the dry needling group received three to seven treatments and CSI group received one injection based on recommendations per standard of care.

### Emerging therapies

- *Autologous tenocyte injection (ATI):* ATI is a cell therapy that has shown encouraging clinical and radiological improvements for the treatment of rotator cuff tendinopathy and chronic lateral epicondylitis. A single prospective study [58] evaluated the effect of ATI in 12 women affected by gluteal tendinopathy who had failed previous non-operative treatments. The results show an incremental clinical improvement both for clinical outcomes and pain reduction especially in the first 12 months, with sustained benefits to 24 months. Overall eight patients were satisfied, two were unsure and two were dissatisfied with their outcomes. No significant improvement from pre- to post-injection was observed in any tendon features at MRI imaging.

## Discussion

GTPS is a multifactorial and often chronic musculoskeletal condition, disproportionately affecting women. Tendinopathy and tears of the gluteus medius and minimus tendons are now recognized as primary causes of GTPS rather than trochanteric bursae pathology. A meticulous clinical examination is required in order to exclude misleading causes of pain, and differential diagnosis should be primarily aimed to exclude intra-articular and extra-articular hip disorders as well as LBP and degenerative lumbar pathologies [27]. Different clinical tests can be performed, but major focus should be given to GT palpation test, resisted hip abduction test, single-leg 30-s stance test, and FABER test in order to increase or decrease the post-test probability of GTPS [24, 26, 59]. US and MRI proved to be valid diagnostic tools, the first being preferred due to its high sensitivity and positive predictive value for gluteal tendinopathy and tendon tears which is comparable or even higher than MRI when in skilled hands. Being accurate, cost-effective, readily available, easily applicable and having the potential of focusing the area of pain allowing for dynamic evaluation and targeted treatment at the same hospital visit makes it the imaging method of first choice for the evaluation of patients with GTPS [2, 26, 60–62]. Conservative treatment and interventional procedures find better application in grade 1 and 2 gluteal tendinopathies, but some (i.e. LR-PRP) could represent a valid choice even for grade 3 gluteal tendinopathies. Although controversy raises about its efficacy, CSI + LA seems more appropriate to gain short-term benefits in the acute phase of GTPS. CSI can be repeated in patients who show a good clinical response after the procedure. Dry needling and HA infiltration could be valid alternatives in those patients for whom CSI + LA is contraindicated (i.e. diabetics). Notably, PRP therapy appears to offer longer-lasting benefits compared to corticosteroids, particularly in patients with symptoms persisting for more than 4 months, and may be effective even in higher-grade tendinopathies Physical therapy, SWT and PRP appear more suitable for achieving long-term benefits in chronic refractory gluteal tendinopathy (symptoms sustained > 4 months). LR-PRP can be proposed as a second interventional procedure after a prior CSI because it appears to not influence the outcome of the LR-PRP injection. Real-time Ultrasound guidance should be preferred over blind injection during interventional procedures in order to precisely visualize and target the desired anatomical structures. Reduction in weight bearing load, activity recommendations, education on load management, weight loss, and avoidance of provoking movements should be encouraged. Overall, GTPS is a chronic and relapsing condition, often requiring a multimodal and individualized management approach. Early diagnosis and standardized, evidence-based treatment protocols are key to optimizing patient outcomes [4]. Nevertheless, some limitations in the current evidence base must be acknowledged. As for grades 1 and 2 tendinopathy, future research should aim to standardise SWT protocols and clearly define energy parameters and session numbers. Comparative studies between SWT and PRP injections are still lacking. Evidence on autologous tenocyte injection remains scarce and based on small-scale, non-randomized studies. The role of percutaneous needle tenotomy needs to be further validated through well-designed trials. Although physical therapy demonstrates long-term effectiveness, the heterogeneity of protocols makes it difficult to determine the superiority of specific exercise types. Additionally, limited evidence is available regarding non-operative approaches for grade 3 tendon tears.

## Conclusions

Greater trochanteric pain syndrome (GTPS) represents a multifactorial and often chronic musculoskeletal condition, primarily driven by gluteal tendinopathy rather than trochanteric bursitis. Accurate clinical examination, supported by imaging—especially high-resolution ultrasound—remains essential for diagnosis. Non-surgical treatments offer a range of effective options, with physical therapy and extracorporeal shock wave therapy showing promising long-term outcomes, particularly in grade 1 and 2 tendinopathy. Corticosteroid injections can provide short-term relief, whereas PRP injections have demonstrated more sustained efficacy, even in cases of grade 3 tendinopathy. Ultrasound-guided procedures are recommended to increase precision and outcomes. Emerging therapies such as autologous tenocyte injections and tendon fenestration warrant further research. Overall, a stepwise, multimodal and individualized approach appears most appropriate for the management of GTPS, while future studies should focus on the standardisation of protocols and long-term comparative efficacy.

## Data Availability

No datasets were generated or analysed during the current study.
